# High-Density Lipoprotein Binds to* Mycobacterium avium* and Affects the Infection of THP-1 Macrophages

**DOI:** 10.1155/2016/4353620

**Published:** 2016-07-19

**Authors:** Naoya Ichimura, Megumi Sato, Akira Yoshimoto, Kouji Yano, Ryunosuke Ohkawa, Takeshi Kasama, Minoru Tozuka

**Affiliations:** ^1^Analytical Laboratory Chemistry, Field of Applied Laboratory Science, Graduate School of Health Care Sciences, Tokyo Medical and Dental University, 1-5-45 Yushima, Bunkyo-Ku, Tokyo 113-8519, Japan; ^2^Department of Clinical Laboratory, Medical Hospital of Tokyo Medical and Dental University, 1-5-45 Yushima, Bunkyo-Ku, Tokyo 113-8519, Japan; ^3^Instrumental Analysis Research Division, Research Center for Medical and Dental Sciences, Tokyo Medical and Dental University, 1-5-45 Yushima, Bunkyo-Ku, Tokyo 113-8519, Japan

## Abstract

High-density lipoprotein (HDL) is involved in innate immunity toward various infectious diseases. Concerning bacteria, HDL is known to bind to lipopolysaccharide (LPS) and to neutralize its physiological activity. On the other hand, cholesterol is known to play an important role in mycobacterial entry into host cells and in survival in the intracellular environment. However, the pathogenicity of* Mycobacterium avium *(*M. avium*) infection, which tends to increase worldwide, remains poorly studied. Here we report that HDL indicated a stronger interaction with* M. avium *than that with other Gram-negative bacteria containing abundant LPS. A binding of apolipoprotein (apo) A-I, the main protein component of HDL, with a specific lipid of* M. avium* might participate in this interaction. HDL did not have a direct bactericidal activity toward* M. avium* but attenuated the engulfment of* M. avium* by THP-1 macrophages. HDL also did not affect bacterial killing after ingestion of live* M. avium* by THP-1 macrophage. Furthermore, HDL strongly promoted the formation of lipid droplets in* M. avium*-infected THP-1 macrophages. These observations provide new insights into the relationship between* M. avium* infection and host lipoproteins, especially HDL. Thus, HDL may help* M. avium* to escape from host innate immunity.

## 1. Introduction 

High-density lipoprotein (HDL), known as antiatherosclerotic lipoprotein, is also involved in innate immunity [1]. For instance, HDL binds to lipopolysaccharides (LPS) and lipoteichoic acid (LTA) derived from microorganisms and neutralizes the physiological activity of these molecules [2, 3]. Additionally, HDL is known to have a bactericidal activity toward* Yersinia enterocolitica *serotype O:3; this activity is mediated by the complement system [4].

*Mycobacterium avium *(*M. avium*) is a species of nontuberculous mycobacteria and causes opportunistic infections in immunocompromised hosts [5]. Although the incidence of* M. avium* infection is increasing worldwide [6], its pathogenicity remains poorly understood unlike that of* M. tuberculosis*. Mycobacterial infection is known to be affected by host lipid metabolism. In particular, cholesterol performs an important function in the invasion and survival of mycobacteria inside macrophages [7–11]. Cholesterol accumulation in the host cell membrane is observed at the entry point of* M. tuberculosis* and of other mycobacteria, and cholesterol depletion inhibits the invasion of cells by mycobacteria. After phagocytosis of mycobacteria, they are enveloped by the cell membrane containing cholesterol-rich domains; tryptophan aspartate-containing coat protein (TACO) is recruited to the phagosomes and prevents the fusion of these organelles with lysosomes [12]. Consequently, the engulfed mycobacteria evade degradation by lysosomes and survive inside the host cell. The invading mycobacteria modulate lipid metabolism in the host cell and promote formation of lipid droplets (LDs), which are mainly composed of neutral lipids such as triacylglycerol and cholesteryl ester [13]. LDs physiologically contribute to lipid storage and lipid metabolism and are available to mycobacteria as a carbon source [10, 14].

Scavenger receptor type B1 (SR-B1), a receptor of apolipoprotein A-I (apoA-I) present in HDL as a main component, is generally known to perform the function of transferring esterified cholesterol from matured HDL into the cytosol in accordance with the cholesterol gradient [15]. SR-B1 was also identified as a nonopsonic phagocytic receptor for mycobacteria because suppression of SR-B1 expression attenuates phagocytosis of mycobacteria [16, 17].

We screened various bacteria for binding with HDL and found that* M. avium* exhibits a stronger interaction with HDL than do other Gram-negative bacilli. The fact that HDL binds to* M. avium* has never been reported and the effect for innate immunity is unclear. The aim of the present study was to elucidate the molecular mechanism of HDL's binding to* M. avium* and the physiological meaning of this interaction.

## 2. Materials and Methods

### 2.1. Bacterial Strains and Growth Conditions

*M. avium* (ATCC 700737) was cultured for 3 weeks in Middlebrook 7H9 broth (Difco) supplemented with the oleic acid-albumin-dextrose complex (OADC; Becton Dickinson). Gram-negative bacteria (isolated at the Clinical Laboratory, Medical Hospital of Tokyo Medical and Dental University) were cultured on trypticase-soy-agar with 5% sheep blood (Becton Dickinson) before use.

### 2.2. Cell Culture, Differentiation, and Infection

The THP-1 cell line was obtained from ATCC (Manassas, VA) and maintained at 2–10 × 10^5^ cells/mL in the RPMI 1640 medium (Sigma-Aldrich) supplemented with 10% heat-inactivated fetal bovine serum (Invitrogen), with a penicillin-streptomycin-L-glutamine solution (Wako), and 1x nonessential amino acids (GIBCO). The THP-1 cells were induced to differentiate into THP-1 macrophages by phorbol myristate acetate (PMA, Sigma-Aldrich) for 72 h. After washing with phosphate-buffered saline (PBS), the THP-1 macrophages were incubated in the serum-free RPMI 1640 supplemented with Nutridoma-SP (Roche) and then infected with* M. avium* (multiplicity of infection [MOI] 20 : 1) with or without HDL (50 *μ*g protein/mL) for 24 h at 35°C in a humidified atmosphere containing 5% CO_2_.

### 2.3. Isolation of HDL and Purification of apoA-I

HDL (1.063–1.210 g/mL) was isolated by means of ultracentrifugation from pooled serum samples obtained from healthy subjects. ApoA-I was purified from HDL according to the previously described method [18]. HDL and apoA-I were dialyzed against PBS and stored at 4°C and −20°C, respectively, until use.

### 2.4. Western Blot Analysis

Bacteria (3 × 10^8^) were washed with PBS and then incubated with 40 *μ*L of normal human serum (NHS), heat-inactivated serum (HIS), HDL, apoA-I, or PBS for 10 min at 37°C. After washing with PBS, the bacteria were mixed with lysis buffer (50 mM Tris-HCl pH 8.0, 150 mM NaCl, 0.1% SDS, and 0.5% sodium deoxycholate). The lysates were then analyzed by electrophoresis on 12.5% SDS-polyacrylamide gels, and the separated proteins were transferred onto polyvinylidene fluoride (PVDF) membranes (Millipore). ApoA-I was detected with a goat anti-apoA-I polyclonal antibody (Academy Bio-Medical Company) followed by a peroxidase- (POD-) conjugated rabbit anti-goat IgG antibody (Medical & Biological Laboratories). Finally, apoA-I was visualized using hydroperoxide and 3,3′-diaminobenzidine as the substrate.

### 2.5. Dot Blot Analysis

This analysis was carried out using a previously described method [19] with a slight modification. Lipids were extracted from the outer cell wall of* M. avium* by means of Folch's extraction procedure [20] and were then spotted onto a nitrocellulose membrane. The membrane was sequentially incubated with 5% (w/v) skim milk and 50 *μ*g/mL HDL in 10 mM Tris-HCl (pH 8.0) containing 140 mM NaCl and 0.1% Tween 20 (TBS-T) for 1 h and 5 h, respectively, at room temperature. The membrane was washed with TBS-T and then incubated with a POD-conjugated anti-apoA-I polyclonal antibody (Cosmo Bio). ApoA-I was visualized as described above.

### 2.6. Thin Layer Chromatography (TLC) and TLC Blot Analysis

The lipids obtained from* M. avium* were spotted onto three Silica Gel 60 plates (Merck Millipore). The TLC plates were simultaneously developed with CHCl_3_/CH_3_OH (95 : 5, v/v). After the run was completed, one of the plates was treated with 20% sulfuric acid solution to visualize the separated lipids, and the other 2 were subjected to TLC blot analysis according to a previously described method [21]. Briefly, the TLC plates were dipped into isopropyl alcohol containing 0.2% aqueous CaCl_2_. The separated lipids were thermally transferred onto a PVDF membrane. One membrane was incubated with 1% bovine serum albumin (BSA) in TBS-T and subsequently incubated with 83 *μ*g/mL of apoA-I in TBS-T at 4°C overnight. The membrane was washed with TBS-T and incubated with the POD-conjugated anti-apoA-I polyclonal antibody (Binding Site). ApoA-I was visualized as described above. To analyze by matrix-assisted laser desorption ionization time-of-flight mass spectrometry (MALDI-TOF MS), the lipid bound to apoA-I was extracted from the other membrane by means of CHCl_3_/CH_3_OH (2 : 1, v/v) according to comparison with the position of the apoA-I spot on the membrane visualized by the TLC blotting described above.

### 2.7. Growth Curve Assay

Live* M. avium* cells (3 × 10^8^/mL) were briefly sonicated to disperse clumps and mixed with the Middlebrook 7H9 broth supplemented with OADC with or without HDL (50 *μ*g protein/mL). Absorbance of the medium at 530 nm was monitored for 144 h.

### 2.8. Phagocytosis Assay Using Flow Cytometry

This assay was carried out according to the previously described method [22, 23]. Briefly,* M. avium* was autoclaved and stained with 0.5 mg/mL fluorescein isothiocyanate (FITC, DOJIN laboratories) in PBS for 30 min. After exhaustive washing, we resuspended the stained* M. avium* at 3 × 10^8^/mL in PBS and then stored the suspension at −80°C until use. THP-1 macrophages (10^6^/well) were cultured with the FITC-conjugated* M. avium* in the presence of HDL (50 *μ*g protein/mL), apoA-I (50 *μ*g/mL), or BSA (50 *μ*g/mL). After quenching extracellular fluorescence using trypan blue (1.2 mg/dL), we fixed the cells with CellFIX (Becton Dickinson) and subjected them to flow cytometric analysis on a Navios flow cytometer (Beckman Coulter). The data were analyzed in the Kaluza software (Beckman Coulter). The FITC-conjugated* E. coli*, which was prepared by the similar method described above, was also analyzed by the phagocytosis assay as the reference.

### 2.9. Assay of Colony-Forming Units (CFUs)

THP-1 macrophages (10^6^/well) were infected with live* M. avium* (MOI 20 : 1) with or without HDL (50 *μ*g protein/mL) for 24 h. The cells engulfing* M. avium* were washed three times with PBS and further cultured in the absence of* M. avium* and HDL for 0, 24, and 48 h. Then, THP-1 macrophages were lysed with PBS containing 0.1% SDS to recover the engulfed* M. avium*. The lysates diluted 100-fold with PBS were cultured on Middlebrook 7H11 agar plates (Difco) supplemented with OADC. CFUs were counted after 3-week incubation at 37°C.

### 2.10. Staining and Quantification of LDs

THP-1 macrophages (5 × 10^5^/well) were infected with live* M. avium* (MOI 20 : 1) with or without HDL (50 *μ*g protein/mL) for 24 h. After being fixed with a 10% formaldehyde solution for 15 min, they are stained with Oil red O (ORO) for 5 min. The cells were counterstained with Mayer's hematoxylin for 2 min in samples to be examined under a light microscope. Lipid bodies were counted for 50 cells. For quantification of LDs amount, infected THP-1 macrophages (5 × 10^5^/well) were stained with only ORO as described above. ORO was then extracted in 500 *μ*L of 100% isopropyl alcohol, and absorbance was measured at 540 nm as described previously [24].

### 2.11. Statistical Analysis

All of the values were shown by the mean ± standard error of the mean (SEM). Statistical analysis was performed by R software. CFUs, the number of LDs, and the others were statistically analyzed by paired Student's *t*-test, Kruskal-Wallis test, and multifactorial analysis of variance (ANOVA) followed by Tukey post hoc tests, respectively.

### 2.12. Ethics Statement

Blood samples were obtained from the healthy donors who had provided written informed consent. The protocol of this study was approved by the Research Ethics Committee of Tokyo Medical and Dental University (decision number 1491).

## 3. Results

### 3.1. Interaction of* M. avium* with HDL through apoA-I

**Figure 1:**
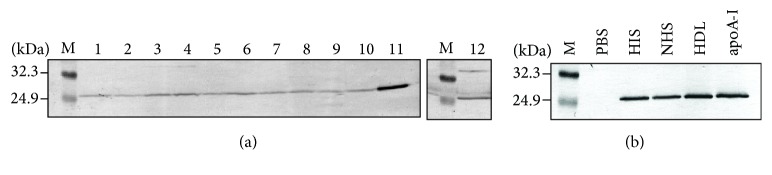
Interaction between bacteria and HDL. (a) The bacterial samples (lane 1:* Pseudomonas aeruginosa*, lane 2:* Aeromonas *species, lane 3:* Klebsiella oxytoca*, lane 4:* Klebsiella pneumoniae*, lane 5:* Serratia marcescens*, lane 6:* Citrobacter koseri*, lane 7:* Pseudomonas aeruginosa*, lane 8:* Enterobacter cloacae*, lane 9:* Acinetobacter baumannii*, lane 10:* Stenotrophomonas maltophilia*, lane 11:* Mycobacterium avium,* and lane 12:* Escherichia coli*) were mixed with normal human serum (NHS). The NHS conjugated bacteria were washed and solubilized by means of lysis buffer. The lysates were separated by 12.5% SDS-PAGE and immunoblotted with anti-apoA-I antibody. The molecular weight markers are also shown (M). (b)* M. avium* was incubated with NHS, heat-inactivated serum (HIS), HDL, and apoA-I for 10 min at 37°C. After washing with PBS,* M. avium* was solubilized and subjected to immunoblotting for apoA-I as described above.

To screen the bacteria for interaction with HDL, some species of bacteria that were incubated with normal human serum (NHS) were analyzed by western blotting with an anti-apoA-I antibody (Figure 1(a)). The apoA-I signal was well visible in the lysate of* M. avium* incubated with NHS but only barely visible in samples from other Gram-negative bacteria. To confirm the direct interaction between apoA-I and* M. avium*, we carried out western blotting of the lysate of* M. avium* incubated with heat-inactivated serum (HIS), NHS, isolated HDL, or purified apoA-I (Figure 1(b)). The apoA-I signal was well visible after incubation not only with NHS but also with the HIS, HDL, and apoA-I, indicating that HDL bound directly to* M. avium* via apoA-I.

### 3.2. ApoA-I Binds to the Outer Cell Wall Lipid of* M. avium*

**Figure 2:**
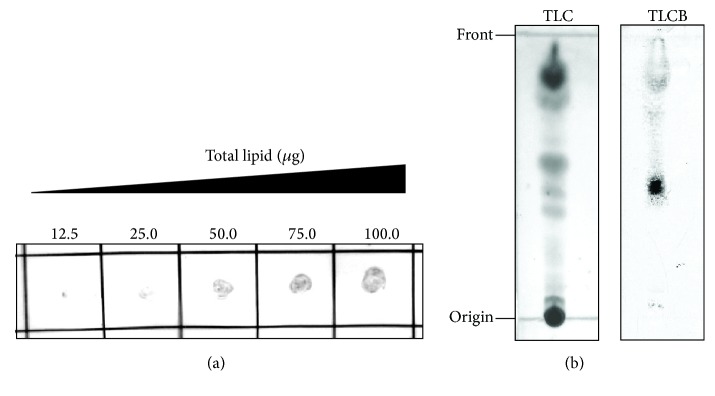
Interaction between lipids of* M. avium* and HDL. (a) The lipids extracted from* M. avium* were spotted in the indicated amount (12.5–100.0 *μ*g) on a nitrocellulose membrane. The membrane was then incubated with HDL (50 *μ*g protein/mL), and HDL bound to the lipids was visualized using an anti-apoA-I antibody. (b) TLC plates with the spotted lipids (150 *μ*g each sample) extracted from* M. avium* were developed using CHCl_3_/CH_3_OH (95 : 5, v/v). One plate was treated with a 20% H_2_SO_4_ solution to visualize the fractionated lipids (TLC). The fractionated lipids on the other plate were thermally transferred onto a PVDF membrane, which was then incubated with HDL (83 *μ*g protein/mL). The lipid that bound to HDL was visualized using an anti-apoA-I antibody (TLCB).

We hypothesized that apoA-I interacts with the abundant lipids on the* M. avium* cell wall because of amphipathic *α*-helices present in the apoA-I molecule. To test this hypothesis, we performed a western blot assay of the lipids extracted from the outer cell wall of* M. avium* (Figure 2(a)). ApoA-I was detected in the spots of the extracted lipids incubated with HDL and the intensity of signals increased with the amount of spotted lipids (at a constant HDL concentration). The TLC blot assay was carried out to determine whether apoA-I binds to a specific lipid molecule of* M. avium* (Figure 2(b)). Several lipid spots on the TLC plate were nonspecifically visualized by a charring reagent (Figure 2(b), left). One of those spots visibly interacted with apoA-I by TLC blot analysis (Figure 2(b), right), indicating that apoA-I formed a complex with this specific lipid of* M. avium*.

The lipids extracted from the apoA-I-positive spot of the TLC blot were analyzed by MALDI-TOF MS (Supplemental Figure 1 in Supplementary Material available online at http://dx.doi.org/10.1155/2016/4353620). Two prominent peaks at* m/z* 1243.8 and* m/z* 1271.8 were observed along with the several peaks with an interval of 28 Da, suggesting that the lipid that bound to apoA-I included a variety of fatty acid moieties.

### 3.3. Analysis of Bactericidal Activity of HDL

**Figure 3:**
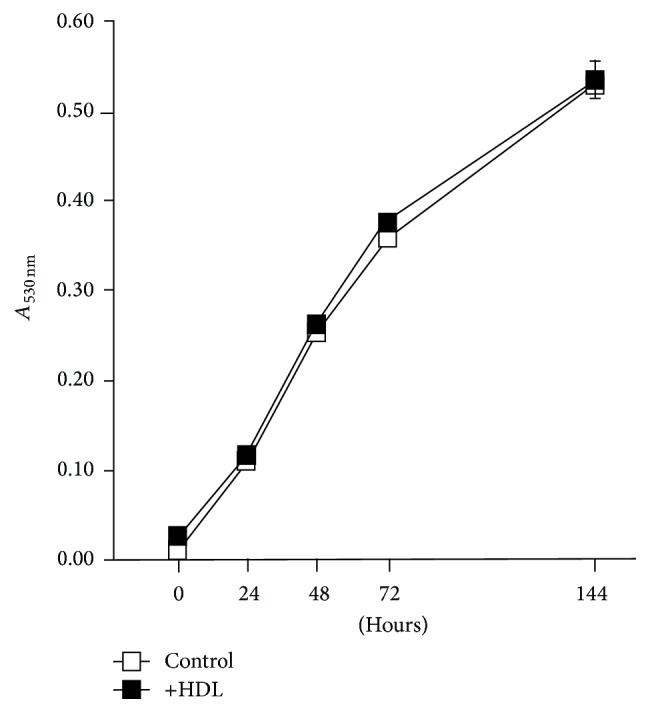
Bactericidal activity of HDL.* M. avium* was cultured with (+HDL; 50 *μ*g protein/mL) or without (control) HDL and absorbance (at 530 nm) of the medium was measured at 0, 24, 48, 72, and 144 h. The values were indicated by mean ± SEM (*n* = 3).

The growth curve analysis of* M. avium* in the presence or absence of HDL was performed to determine whether HDL directly kills* M. avium* (Figure 3). No significant difference was observed in the absorbance of the medium with or without HDL for 6 days. This finding indicated that HDL itself had no bactericidal activity toward* M. avium*.

### 3.4. Effects of HDL on Engulfment of* M. avium* by THP-1 Macrophages

**Figure 4:**
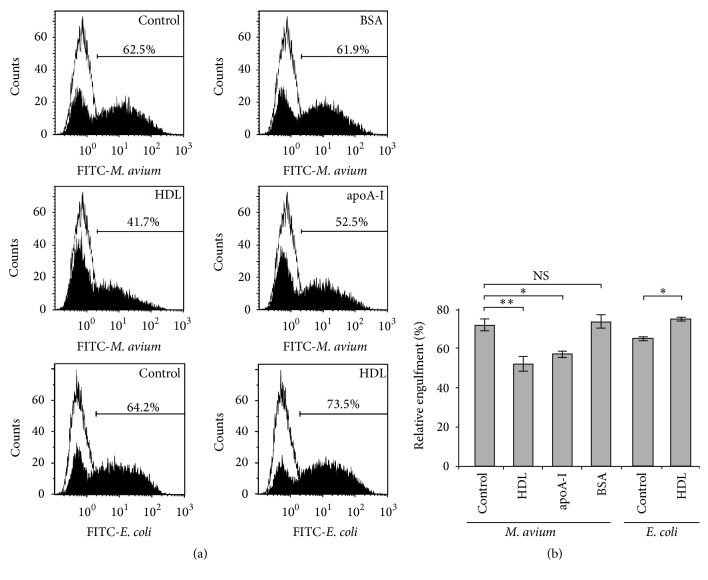
Effects of HDL on the engulfment of* M. avium* by THP-1 macrophages. (a) THP-1 macrophages (10^6^/well) were cultured with FITC-conjugated* M. avium* or* E. coli* in the presence or absence (control) of HDL (50 *μ*g protein/mL), apoA-I (50 *μ*g/mL), or BSA (50 *μ*g/mL) for 24 h. After extensive washing and quenching of extracellular bacteria, the cells were subjected to flow cytometric analysis. Representative scatter profiles are presented. (b) The percentages of FITC-positive cells compared to the control using FITC-*M. avium* or FITC-*E. coli* were quantitatively compared. The values were indicated by mean ± SEM (*n* = 3, ^*∗*^ *P* < 0.05, ^*∗∗*^ *P* < 0.01). NS: not significant.

To find out whether HDL participates in the nonopsonic phagocytosis of* M. avium* by THP-1 macrophages, we used flow cytometry to analyze the effect of HDL on the number of THP-1 macrophages engulfing FITC-conjugated* M. avium* (Figure 4(a)). The percentage of FITC-positive cells decreased with the addition of HDL and apoA-I but did not change significantly after addition of BSA. The percentage of FITC-positive cells which is obtained using FITC-conjugated* E. coli *indicated a similar result to that of FITC-conjugated* M. avium*; however the addition of HDL did not decrease the percentage of FITC-positive cells. When the number of FITC-positive cells of control samples was set to 100%, HDL and apoA-I decreased the number of FITC-positive cells to 68.7%  ± 1.5% and 83.3%  ± 0.4%, respectively (Figure 4(b)). BSA did not affect the engulfment of* M. avium* by THP-1 macrophages (101.8%  ± 2.0%). In contrast, FITC-positive cells did not decrease, if anything increased, in the case of FITC-conjugated* E. coli*.

### 3.5. Effects of HDL on Bactericidal Activity of THP-1 Macrophages

**Figure 5:**
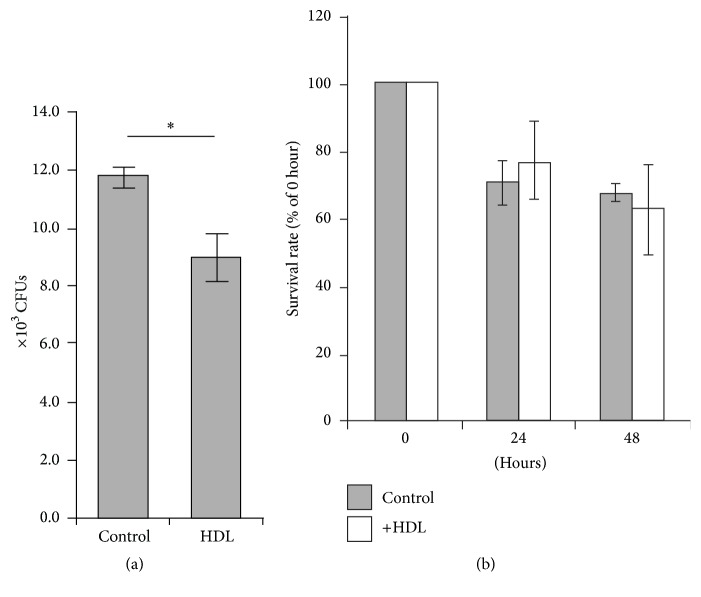
Effects of HDL on the viability of* M. avium* engulfed by THP-1 macrophages. (a) THP-1 macrophages (10^6^/well) were infected with live* M. avium *in the presence or absence (control) of HDL (50 *μ*g protein/mL) for 24 h.* M. avium* was recovered from lysed THP-1 macrophages for an assay of colony-forming units (CFUs; 3 weeks culture). (b) A part of infected THP-1 macrophages was further cultured for 24 and 48 h in a fresh medium. CFUs were also determined as described above. The data were indicated as the percentages against CFUs obtained without further cultivation. The values were indicated by mean ± SEM (*n* = 3, ^*∗*^ *P* < 0.05).

Assay of CFUs was performed to assess the number of live* M. avium* inside THP-1 macrophages by means of* M. avium* recovered from the lysed THP-1 macrophages (Figure 5(a)). The results that were expressed in CFU/well were (8.5 ± 1.2) × 10^4^ and (11.7 ± 0.6) × 10^4^ (mean ± SE) in the presence and the absence of HDL, respectively. This observation is consistent with the result obtained by the flow cytometric analysis (Figure 4), namely, that HDL attenuated the engulfment of* M. avium* by THP-1 macrophages. To assess the survival rate of* M. avium* inside THP-1 macrophages, the infected THP-1 macrophages were further cultured in the absence of* M. avium* and HDL for 24 and 48 h. The number of CFUs per well indicated a tendency to decrease in a time-dependent manner (Figure 5(b)). However, no significant difference was observed in the decreasing profiles between the presence and the absence of HDL, suggesting that the coexistence of HDL could not affect bacterial killing after ingestion by THP-1 macrophage.

### 3.6. Effects of HDL on LDs Formation

**Figure 6:**
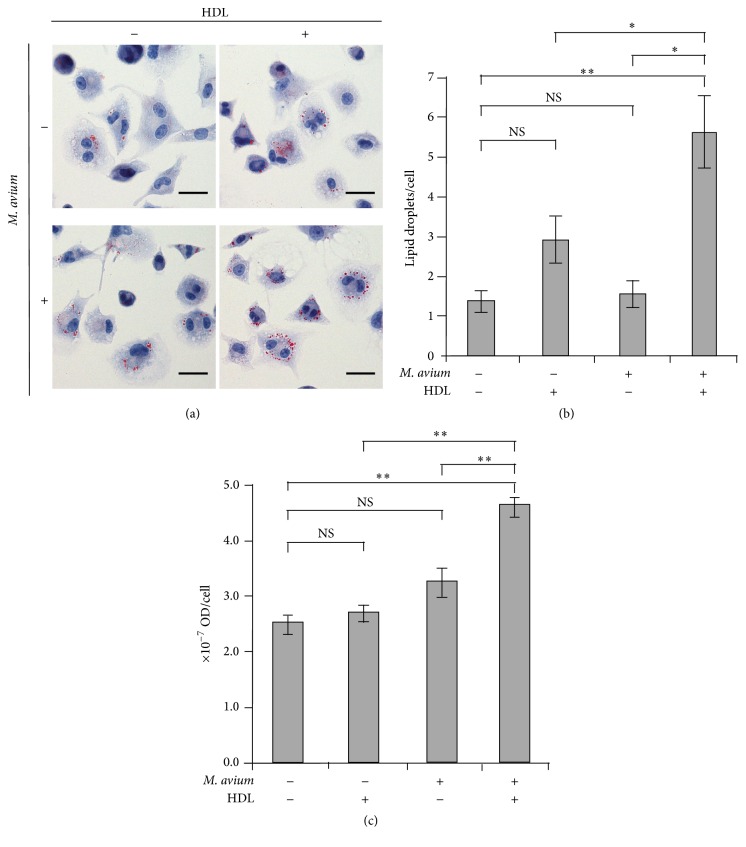
High-density lipoprotein (HDL) promotes a formation of THP-1 macrophage-derived foam cells during* M. avium* infection. THP-1 macrophages were infected by* M. avium* with (+) or without (−) HDL. (a) Representative light microscopic images of the cells stained with Oil red O (ORO) and hematoxylin are shown. The scale bar is 25 nm. (b) The numbers of lipid droplets (LDs) per cell were calculated by counting of those number for 50 cells. (c) The amount of LDs was also quantitatively analyzed by measuring absorbance (at 540 nm) of ORO extracted from the cells. The values were indicated by mean ± SEM (*n* = 3, ^*∗*^ *P* < 0.05, ^*∗∗*^ *P* < 0.01). NS: not significant.

After the infection of THP-1 macrophages with live* M. avium*, LDs inside the cells were stained with Oil red O (ORO) (Figure 6(a)). The numbers of LDs per cell were significantly increased by the coexistence of live* M. avium* and HDL (Figure 6(b)). LDs amounts were also estimated by the absorbance at 540 nm of the extracted ORO (Figure 6(c)). The existence of HDL or* M. avium* alone indicated no significant effect in the quantity of ORO compared with the control. However, the coexistence of HDL and* M. avium* caused an increase in the LDs amount.

## 4. Discussion

In this study, we found that HDL binds to* M. avium *via the interaction between apoA-I (the main structural component of HDL) and a specific lipid molecule of* M. avium*. The specific lipid could be extremely nonpolar considering its behavior during TLC analysis. Although a structure of the specific lipid molecule was analyzed by MALDI-TOF-MS/MS (data not shown), we failed to identify it among the known lipid molecules of* M. avium.*

It should be noted that* M. avium* causes inflammation and increases permeability of blood vessels, increasing the chance of interaction with leaked HDL. If this specific lipid of* M. avium* plays a role in host pathogenesis, HDL may neutralize the activity of this lipid, just as in the case of LPS and LTA. It could be important to identify the lipid molecule in order to elucidate a participation of HDL in* M. avium* infection.

We explored the influence of HDL's binding to* M. avium *on an innate immunity in experiments with THP-1 macrophages. HDL does not directly kill* M. avium*, but the presence of HDL significantly decreases the number of THP-1 macrophages engulfing* M. avium*, and HDL indicated no effect on bacterial killing after ingestion. It suggests that HDL allows* M. avium* to escape engulfment by THP-1 macrophages. SR-B1, known as an apoA-I receptor, also plays a role in nonopsonic phagocytosis of mycobacteria [16, 17]. According to these observations, we can hypothesize that HDL competitively inhibits the binding of* M. avium* to SR-B1. In this study, we did not analyze SR-B1, and further research is needed to assess the interaction between* M. avium* and HDL, including the participation of SR-B1.

When mycobacteria are recognized by Toll-like receptors 2 or 6 (TLR2/6), LDs formation is enhanced by TLR signaling [25, 26]. Moreover, some researchers proposed a mechanism behind the enhancement of LDs formation during mycobacterial infection: upregulation of a series of scavenger receptors including SR-B1 [27]. In our study,* M. avium* infection did not apparently upregulate LDs formation under serum-free condition; however, the coexistence of HDL increased the number and the amount of LDs in THP-1 macrophages, suggesting that HDL-cholesterol is utilized as a raw material for LDs formation. Two mechanisms may explain this observation. According to one mechanism, HDL-cholesterol is internalized together with* M. avium* via phagocytosis. According to the other, although approximately 30% of the engulfment of* M. avium* by THP-1 macrophages was attenuated by the coexistence of HDL (Figure 4), the HDL-cholesterol influx is enhanced by upregulation of SR-B1 in THP-1 macrophages engulfing* M. avium*. Consequently, TLR signaling and HDL-cholesterol influx via SR-B1 may promote LDs formation in the presence of both* M. avium* and HDL. Further research is needed to determine the actual mechanism.

On the basis of the present study, we believe that HDL plays a crucial role in the infection of THP-1 macrophages by* M. avium*. Identification of the lipid molecule of* M. avium* that binds to HDL via apoA-I and the analysis of the expression of the scavenger receptor, SR-B1, in an upcoming project, should shed some light on the pathogenesis of* M. avium *infections and on the relevant immune response.

## 5. Conclusion

HDL affects* M. avium* infection through the binding between apoA-I, the main component of HDL, and a specific lipid of* M. avium*. This interaction may enable* M. avium *to escape from innate immunity THP-1 macrophages.

## Supplementary Material

The specific lipid bound to apoA-I was obtained from the TLC blot and mixed with a matrix solution: 2,5-dihydroxy benzoic acid (DHB; Sigma-Aldrich) in 50% acetonitrile containing 0.1% trifluoroacetic acid. MALDI-TOF MS spectra were obtained using the reflectron and positive ion mode using ultrafleXtreme TOF/TOF instrument (Bruker). Two prominent peaks at m/z 1243.8 and m/z 1271.8 were observed along with the several peaks with an interval of 28 Da, suggesting that the specific lipid included a variety of fatty acid moieties.
