# Supplementary material for: High-Density Lipoprotein Binds to Mycobacterium avium and Affects the Infection of THP-1 Macrophages
Source: J Lipids. 2016 Jul 19;2016:4353620. doi: 10.1155/2016/4353620 (PMC4969507; doi:10.1155/2016/4353620)

Supplementary Figure 1. MS analysis for apoA-I binding lipid.The lipid obtained from the TLC blot was mixed with a matrix solution: 2,5-dihydroxy benzoic acid (Sigma-Aldrich) in 50% acetonitrile containing 0.1% trifluoroacetic acid. MALDI-TOF MS spectra were obtained using the reflectron and positive ion mode using ultrafleXtreme TOF/TOF instrument (Bruker).

Supplemental Figure 1

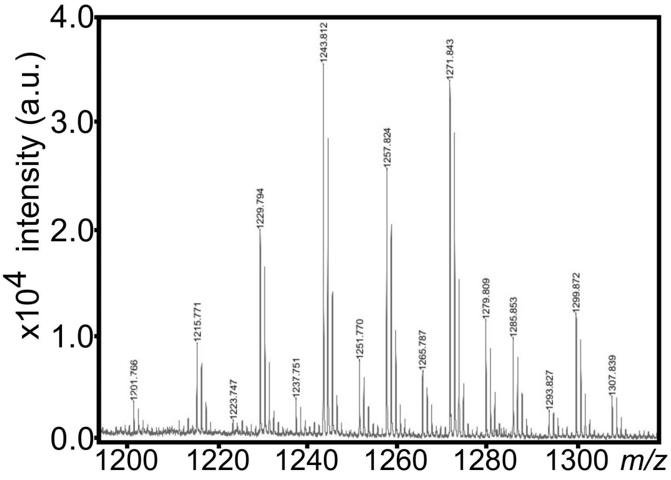

Supplement: Supplementary file 1 — The specific lipid bound to apoA-I was obtained from the TLC blot and mixed with a matrix solution: 2,5-dihydroxy benzoic acid (DHB; Sigma-Aldrich) in 50% acetonitrile containing 0.1% trifluoroacetic acid. MALDI-TOF MS spectra were obtained using the reflectron and positive ion mode using ultrafleXtreme TOF/TOF instrument (Bruker). Two prominent peaks at m/z 1243.8 and m/z 1271.8 were observed along with the several peaks with an interval of 28 Da, suggesting that the specific lipid included a variety of fatty acid moieties. [file 4353620.f1.pdf]
